# Unveiling the potential of Muscadine grape Skin extract as an innovative therapeutic intervention in cancer treatment

**DOI:** 10.1016/j.jff.2024.106146

**Published:** 2024-04-06

**Authors:** Sarah Otun, Ikechukwu Achilonu, Valerie Odero-Marah

**Affiliations:** a School of Molecular and Cell Biology, Faculty of Science, Protein Structure-Function and Research Unit, University of the Witwatersrand, Braamfontein, Johannesburg, South Africa; b Center for Urban Health Disparities Research and Innovation, Department of Biology, Morgan State University, Baltimore MD 21251, United States

**Keywords:** Anti-Cancer, Antioxidants, Muscadine grape skin extract, Phytochemicals, Plant-derived medicinal extracts, Toxicological properties

## Abstract

The use of muscadine grape extracts (MGSE). in cancer treatment has gained attention due to its distinctive composition of polyphenols and antioxidants. This review analyses the reported anti-cancer properties of MGSE. The study commences by reviewing the phytochemical composition of MGSE, highlighting the presence of resveratrol and ellagic acid. Furthermore, the review underscores the mechanism of action of these active compounds in MGSE in combating cancer cells. The anti-cancer potential of MGSE compared to other plant extracts is also discussed. In addition, it highlights MGSE’s superiority and distinct phytochemical composition in preventing cancer growth by comparing its anti-cancer compounds with those of other anti-cancer medicinal plants. Lastly, the combinatory approaches of MGSE with traditional cancer therapies, its safety, and its possible side effects were highlighted. This work provides an understanding of the anti-cancer properties of MGSE, positioning it as a valuable and unique challenge within the field of cancer therapy.

## 1. Introduction

Despite significant advancements in our understanding of cancer and its treatment, this disease remains a pervasive global concern, impacting a vast number of individuals across the globe (Zhu et al., 2023). The anticipated increase in global cancer cases, projected to rise from 12.7 million in 2008 to 20.3 million by 2030, is primarily attributed to the ongoing growth and aging of the worldwide population (Hack et al., 2019). According to the American Cancer Society, as of Jan 1, 2022, more than 18 million Americans were living with cancer, with the majority having received their diagnosis years earlier and exhibiting no present symptoms (American Cancer Society, 2022). However, their projections indicate that by the end of 2023, the United States is expected to see over 1.9 million new cancer cases, resulting in an estimated 609,820 cancer-related deaths. This equates to an average of approximately 1,670 deaths per day, establishing cancer as the second most prevalent cause of death in the United States, trailing closely behind heart disease (American Cancer Society, 2022).

The latest data from the American Cancer Society indicates that progress and challenges exist in the battle against cancer. While there is an overall decrease in mortality rates, with a projected 1.5 % decline between 2019 and 2020, certain cancers such as prostate, breast, and uterine corpus are becoming more prevalent-especially among specific racial groups where they exhibit significant inconsistencies of death tolls (Siegel et al., 2023). Nevertheless, notable improvements in medical procedures have led to rapid declines in Leukaemia, kidney, etc, despite constant or increasing growth rates (American Cancer Society, 2022). Aside from this, development has also been observed in early twenties women whose cervical cancer occurrence rate suffered due to the Human Papillomavirus vaccine providing opportunities towards downsizing HPV-associated types, amongst others (Aretha, 2021). In its entirety, though some successful strides have been taken, regard must still be paid to growing occurrences cases as good disparities while paying heed to research initiatives coupled with accessibility criteria from potential treatments (incredibly innovative) issues which underpins their eradication effort so far made (Cao et al., 2020).

However, despite its ranking as the second leading cause of death, cancer’s development is a complex process characterized by multiple stages that unfold over an extended period, often spanning several years or even decades (American Cancer Society, 2022; Mahvi et al., 2018). Hence, inhibiting one or more of these stages could impede cancer progression, thus allowing natural agents to serve as preventative and intervention mediators in specific instances (Bandi et al., 2021). Therefore, it is crucial to continually pursue innovative strategies that can effectively supplement existing cancer treatments, thus enhancing patient outcomes and alleviating the burden of this disease (M. Huang et al., 2021).

In recent years, significant progress has been made in investigating natural compounds and their extracts as potential agents for combating cancer (Bhuia et al., 2023; M. Huang et al., 2021; Varghese & Dalvi, 2021). One area that has garnered considerable attention in this field of oncology is MGSE, obtained from the Muscadine Grape (*Vitis rotundifolia*). MGSE contains a high concentration of phytochemicals, including resveratrol, ellagic acid, quercetin, and anthocyanins, which have been extensively studied for their antioxidant and anti-inflammatory effects (Dansby, 2007). This review aims to examine the therapeutic efficacy of MGSE in cancer treatment thoroughly. Through an in-depth analysis of its phytochemical composition, mechanisms of action, and a comprehensive review of existing preclinical and clinical evidence, we aim to provide insight into the potential of MGSE as an innovative therapeutic agent in the battle against cancer. Furthermore, we report the possibility of integrating MGSE with traditional cancer therapies and analyze the safety considerations associated with its use.

## 2. Muscadine grape Skin Extract: Composition and characteristics

The Muscadine grape, scientifically known as *Vitis rotundifolia*, distinguishes itself from the more common red grapes often employed for red wine production by its notable resveratrol content (Stewart et al., 2023). Indigenous to the Southeastern region of the United States, Muscadine grapes naturally thrive from Delaware to the Gulf of Mexico and extend westward from Missouri to Texas (Hickey et al., 2019). It is worth noting that the phytochemical composition of Muscadine grapes sets them apart from other grape types due to their elevated levels of resveratrol, anthocyanin, ellagic acid, and ellagic acid precursors (Narduzzi et al., 2015). Anthocyanins primarily contribute to these grapes’ red and purple coloration (Yuzuak & Xie, 2022). These phytochemicals exhibit potent antioxidant properties (Mendonca et al., 2019) and demonstrate anti-cancer effects through various mechanisms, including hindering the formation of DNA adducts induced by carcinogens (W. Li et al., 2022), impeding DNA synthesis in breast cancer cells (Collard et al., 2020), and retarding angiogenesis in specific tumor types (Burton et al., 2015; God et al., 2007; W. Li et al., 2022). Furthermore, previous research has indicated that aqueous extracts derived from the whole muscadine berry can impede the activities of matrix metalloproteinases (MMP2 and MMP9), enzymes associated with tumor metastasis (Hudson et al., 2007). Specifically, the skins of this fruit serve as a valuable reservoir of phytochemical compounds that have garnered considerable interest due to their potential therapeutic properties, particularly in the context of cancer treatment (Singh et al., 2015).

### i. Phytochemical profile

The therapeutic potential of MGSE can be attributed to its abundant and diverse phytochemicals (Stewart et al., 2023). These bioactive compounds within MGSE serve as the foundational elements behind their remarkable antioxidant and anti-cancer properties (MENDONCA et al., 2019a). Table 1 is a comprehensive list of the most prominent phytochemical constituents found in MGSE. Understanding these phytochemical compositions of MGSE is paramount in elucidating its mechanisms of action against cancer. These compounds can selectively target various aspects of cancer initiation and progression (Dansby, 2007; MENDONCA et al., 2019a).

### ii. Antioxidant properties

The exceptional antioxidant properties of MGSE distinguish it from natural compounds (Dansby, 2007). The diverse range of phytochemicals present in this substance contributes to its various properties, enabling it to combat oxidative stress and its associated damage effectively. An in-depth comprehension of MGSE’s antioxidant capacity is pivotal to grasping its role in cancer prevention and treatment. For instance, oxidative stress occurs when there is an imbalance between the production of free radicals and the presence of antioxidants within the human body (Sailaja Rao et al., 2011). Free radicals, which are high reactivity molecules, pose a risk of causing harm to various cellular components, including DNA, proteins, and lipids (Juan et al., 2021). This damage can lead to cellular dysfunction and potentially expedite the development of cancer (Kehrer & Klotz, 2015). MGSE, enriched with abundant polyphenols such as resveratrol and quercetin, stands out for its significant capacity to mitigate the detrimental effects of free radicals, thereby reducing the risk of oxidative damage (Akbari et al., 2022).

Furthermore, preserving cellular integrity is a critical function attributed to the antioxidants found in MGSE (Stewart et al., 2023). These antioxidants play a vital role in protecting cellular components from harm induced by oxidative stress, supporting the normal function of cells (Zulfiqar & Ashraf, 2022). This protective effect extends to preventing genetic alterations that could trigger mutations in otherwise healthy cells (Ding et al., 2020). Recent studies suggest that the antioxidants present in MSGE can influence the modulation of signaling pathways associated with cancer progression (Filip et al., 2013; Singh et al., 2016). The immune-boosting attributes of antioxidants in MGSE, such as quercetin, have been scientifically demonstrated to enhance immune cell activity (Manso et al., 2021), thereby supporting the body’s innate defense mechanisms against cancer. MGSE’s substantial antioxidant properties render it a promising contender for cancer prevention and supplementary therapeutic interventions. By mitigating the detrimental impacts of oxidative stress, MGSE establishes a less favorable environment for the initiation and progression of cancer (Iqbal et al., 2021). Hence, alongside its additional bioactive properties stemming from phytochemicals, MGSE emerges as a potentially valuable natural asset in the fight against cancer (MENDONCA et al., 2019b).

### iii. Anti-Inflammatory properties

The recognition of chronic inflammation’s role in the initiation and progression of various cancer types is steadily growing (Shah & Itzkowitz, 2022; Sharma et al., 2019). Therefore, the investigation of MGSE has garnered significant interest in cancer prevention and treatment owing to its anti-inflammatory properties. The association between inflammation and cancer is defined by chronic inflammation’s capacity to facilitate the proliferation and survival of cancer cells (Piotrowski et al., 2020). Inflammatory processes have the potential to reduce genetic mutations, stimulate angiogenesis (the process of generating new blood vessels to support tumors), and enhance the growth of malignant cells (Aguilar-Cazares et al., 2019; Lugano et al., 2020). MGSE’s potential to counteract these processes can be attributed to its anti-inflammatory properties.

The modulation of inflammatory signaling pathways has been investigated with the phytochemicals found in MGSE, including resveratrol, quercetin, and ellagic acid (R. Li et al., 2017). A notable example is the inhibitory impact of resveratrol on the NF-κB pathway, a crucial regulator of inflammation often dysregulated in the context of cancer (Krajka-Kuźniak & Baer-Dubowska, 2021). MGSE’s ability to mitigate the inflammatory response may be linked to its capacity to reduce NF-κB activity. Beyond its direct influence on inflammatory pathways, MGSE may impact the immune system’s inflammatory response (Krajka-Kuźniak & Baer-Dubowska, 2021). By promoting a balanced immune response, MGSE can establish a less favorable environment for cancer development.

## 3. Muscadine grape Skin extract vs other plant-derived medicinal extracts

The unique and exclusive compounds found in MGSE are the key to its potent antioxidant and anticancer effects and a novel area of research. While some of the phytochemicals discovered in MGSE can also be found in other plant sources, the specific components exclusive to Muscadine grapes are less abundant elsewhere, making this extract a fascinating and unexplored subject of study (Wei et al., 2017). For instance, Ellagic acid, a phytochemical with potent antioxidant and anticancer properties, is found in large quantities in Muscadine grapes, especially in the skin, giving the fruit vivid color and health advantages (A. Marshall, 2012). Numerous studies have been conducted on Ellagic acid’s ability to prevent DNA damage caused by free radicals, initiate apoptosis, and obstruct the growth of cancer cells (Akumadu et al., 2020; Duan et al., 2020; Lee & Talcott, 2002; Marshall-Shaw et al., 2014). Furthermore, ellagitannins, a distinct class of polyphenols that precede ellagic acid, are abundant in Muscadine grapes. Ellagic acid is released when ellagannins are hydrolyzed in the stomach, and this is when it starts to work (Sandhu & Gu, 2010). Although these substances are uncommon in other fruits and vegetables, they are abundant in MGSE. While the uniqueness of substances like ellagic acid and ellagitannins in Muscadine grapes is apparent, this extract holds great promise in cancer prevention and treatment, inspiring us as scientists and researchers to delve deeper into its potential. Table 2 compares the presence of several anti-cancer compounds in MGSE with those found in other plant extracts, such as ginger, garlic, ginseng, turmeric (curcumin), green tea, and ginseng.

**Fig. 1.**
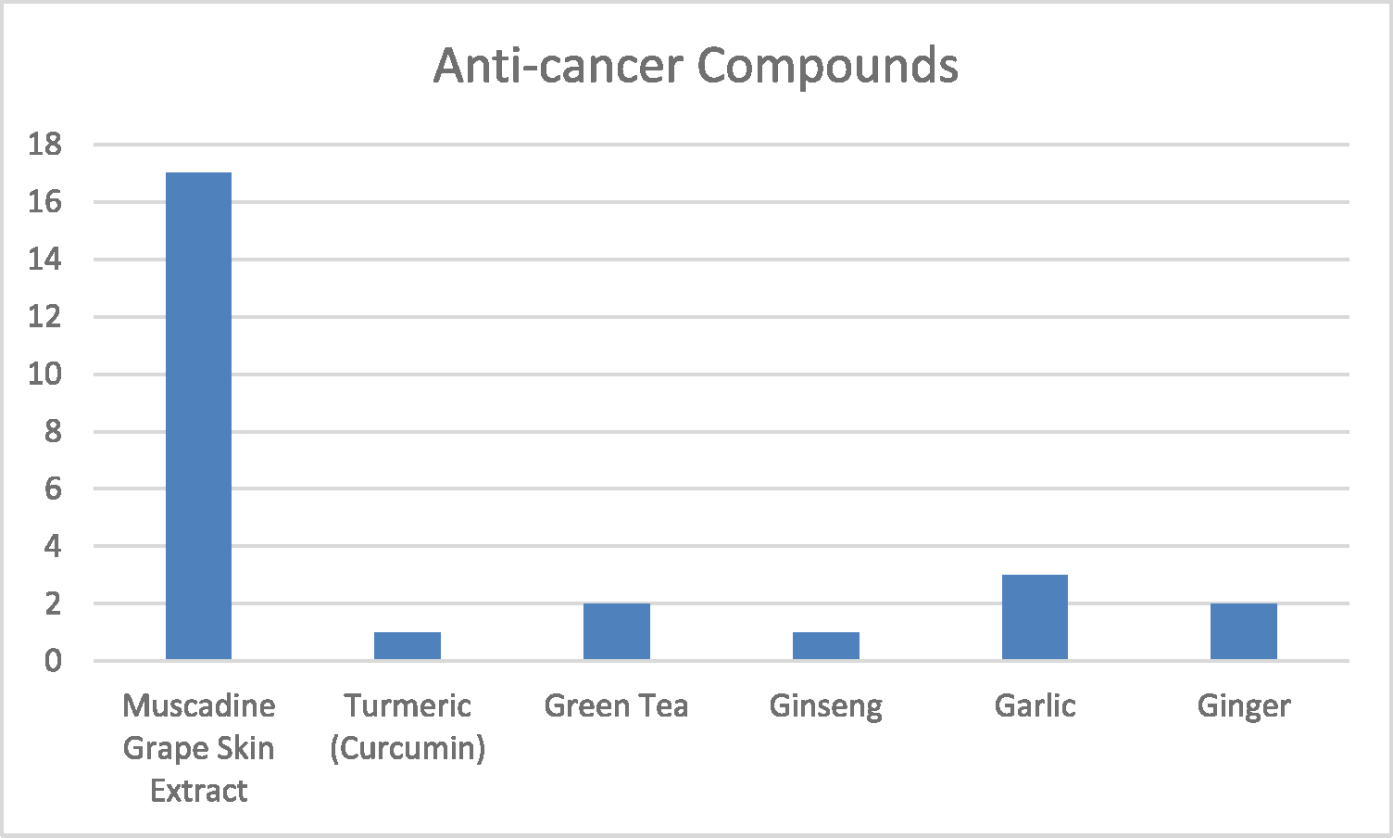
Comparison of the number of Anticancer compounds in Plant-Derived medicinal extracts.

As shown in Table 2, MGSE contains a unique blend of anti-cancer substances, such as ellagic acid, quercetin, OPCs, and ellagitannins. Green tea is high in catechins and EGCG, whereas turmeric (curcumin) predominantly includes curcumin. Garlic is renowned for allicin and alliin, gingerol and shogaol, and ginsenosides in ginseng. Every dietary ingredient generated from plants that can fight cancer has its unique combination of chemicals. The superiority of MGSE in the fight against cancer, when compared with other plant-derived extracts, is also shown in Fig. 1. Fig. 1 illustrates the richness of MGSE composition by contrasting its 17 compounds with extracts that include fewer compounds (usually 2 or 3). MGSE’s higher anti-cancer benefits may be attributed to its more extensive diversity of bioactive chemicals, as shown in Fig. 1, compared to other plant-derived extracts, offering significant insights into their respective advantages and possible therapeutic uses.

This review is essential because it thoroughly assesses Muscadine Grape Skin Extract’s (MGSE) distinct anticancer qualities in relation to other plant-derived anticancer dietary ingredients. It illuminates the possible advantage of MGSE in the fight against cancer by dissecting its unique phytochemical makeup and effectiveness. Comprehending the processes and substances in MGSE that underpin its anticancer properties yields significant insights for forthcoming investigations and clinical implementations. Thus, this review is invaluable for researchers, medical professionals, and anybody looking for complementary or alternative cancer therapies made from natural sources.

## 4. Muscadine grape Skin extract in cancer research

Exploring the potential of natural compounds and botanical extracts as cancer-fighting agents has opened a promising field of academic investigation (Singla et al., 2021). The origins of using grapes and their derivatives for medicinal purposes can be traced across various civilizations and eras.(Balachandran & Govindarajan, 2005; Soleas et al., 1997). Grapes have been cherished for their culinary pleasures and revered for their perceived medicinal benefits throughout history (Cosme et al., 2022). The historical use of grapes, particularly the distinctive Muscadine variety, as therapeutic agents presents an intriguing context for the current investigation of MGSE in cancer research.

The medicinal properties of grapes were recognized by ancient civilizations, including the Greeks and Egyptians (Bertelli & Das, 2009). Grapes, grape leaves, and grape juice were employed to treat various ailments, from digestive disorders to skin conditions (Kersh et al., 2023). The consumption of grapes has often been linked to attributes of vitality and longevity (Bertelli & Das, 2009). Similarly, wine as a medicinal substance holds significant historical significance, as it was widely utilized in ancient medical traditions (Liang et al., 2021). Wine, derived from the fermentation of grapes, occupied a prominent position within early medical practices (Xu et al., 2020). Hippocrates, widely recognized as the founder of modern medicine, notably believed that moderate wine consumption could promote healing and restore one’s health (McGovern et al., 2003).

In subsequent centuries, the progression of scientific knowledge enabled the precise identification of phytochemicals in grapes that contribute to their health benefits, marking a sharp contrast with the historical approach that leaned on empirical observations. The discovery of resveratrol, ellagic acid, quercetin, and anthocyanins within Muscadine grapes has provided insight into the specific compounds that contribute to their therapeutic properties (Marshall-Shaw et al., 2014). Hence, the historical significance attributed to grapes, coupled with the current knowledge regarding their phytochemical makeup, has facilitated the systematic investigation of MGSE in cancer research.

A plethora of studies conducted both in laboratory settings (in vitro) and in living organisms (in vivo) have shed light on the impacts of MGSE on cancer cells and the surrounding tumor microenvironment (Cháirez-Ramírez et al., 2021; Paller et al., 2018; Peterson et al., 2022; Singla et al., 2021). For instance, according to the research conducted by Hudson et al. (2007) on prostate cancer growth inhibition by MGSE and resveratrol through different mechanisms, The researchers performed a comparison study to evaluate the anti-cancer effects of MGSE without resveratrol versus resveratrol. This analysis was performed on primary cultures of normal prostate epithelial cells (PrEC) as well as various prostate cancer cell lines (RWPE-1, WPE1-NA22, WPE1-NB14, and WPE1-NB26), which represent distinct stages of prostate cancer progression. The MSKE treatment inhibited all prostate cancer cell lines that transformed. However, no such effects were seen on PrEC cells (Hudson et al., 2007). Other notable studies on the anti-cancer efficacy of MGSE are further discussed.

## 5. Muscadine grape extract as a potential treatment for Triple-Negative breast cancer

Triple-negative breast cancer (TNBC) is an aggressive and complex subtype characterized by the absence of estrogen receptor, progesterone receptor, and human epidermal growth factor receptor 2 (HER2) expression, with a disproportionate impact on African American women. Recent studies have explored the potential of MGE in addressing TNBC. Mendonca et al.’s research delved into the antioxidant and anti-cancer attributes of MGE in TNBC cells, emphasizing mitigating racial disparities in TNBC incidence (Mendonca et al., 2019). Notably, MGE displayed enhanced antioxidant capacity and cytotoxicity in African-American cell lines compared to Caucasian cell lines. This suggests a potential avenue for reducing racial differences in TNBC outcomes, particularly in African-American women.

Similarly, Collard and his research group explored the impact of a polyphenol-enriched extract from muscadine grapes on TNBC tumor formation in nude mice (Collard et al., 2020). Their research demonstrated that MGE effectively reduced tumor size and the expression of proliferation-associated markers, such as Ki67 and cyclin D1, thus promising to inhibit TNBC progression. In an additional study, Collard et al. examined the effects of MGE on TNBC metastasis and gut microbiota (Collard et al., 2019). MGE significantly reduced metastatic proliferation and cancer-associated fibroblasts in the lungs and liver. Furthermore, MGE induced modifications in the gut microbiome, resulting in an augmentation of both richness and diversity, accompanied by a notable transition towards a more advantageous bacterial composition. The observed rise in the abundance of butyrate bacteria and the corresponding increase in butyrate concentrations indicate a potential anti-inflammatory mechanism that might impede the spread of TNBC. These findings suggest that MGE may be a valuable therapeutic option for inhibiting TNBC metastatic and addressing this aggressive cancer subtype (Collard et al., 2019).

The investigation conducted by Burton et al. (2019) examined CCAAT-displacement protein/cut homeobox transcription factor 1 (CUX1) in TNBC and its role in suppressing estrogen receptor-alpha (ER-α) (Burton et al., 2019). Elevated CUX1 and cathepsin L (Cat L) levels were observed in TNBC tissues and cell lines. Research findings suggest that CUX1 directly interacts with the ER-α promoter in TNBC cells, thus suppressing its transcription. Notably, the administration of MSKE, rich in anthocyanins or a Cat L inhibitor, effectively inhibited CUX1 and Snail expression, reducing cell invasion and increasing apoptosis. Furthermore, Mackert and colleagues explored HER2-positive breast cancer, investigating how MGE could exert inhibitory effects on trastuzumab-sensitive and trastuzumab-resistant HER2-positive cells (Mackert et al., 2019).

Additionally, the research team sought to establish whether MGE had any synergistic effects when combined with trastuzumab (TRZ). MGE suppressed cell growth when combined with trastuzumab, offering a potential complementary treatment. This inhibitory effect was contingent on the duration of treatment and the dosage of MGE.

Furthermore, Collard and colleagues examined the cytotoxicity of stilbene-rich muscadine grape berry extracts against various cancer cell lines, demonstrating superior effectiveness to pure resveratrol (Collard et al., 2019). These extracts induced substantial cell mortality, whereby a concentration as low as 1 μg/mL led to a 50–80 % decrease in cell viability over 72 h. In contrast, a 50 μg/mL concentration of resveratrol was necessary to achieve a similar outcome. In addition, the extracts containing high levels of stilbene exhibited enhanced regulation of tumor development and suppression genes in liver cancer cells, surpassing the effects of resveratrol alone. The present work emphasizes the possible synergistic effects of stilbenes found in muscadine grape berries, presenting a prospective path for developing more effective anti-cancer medicines.

## 6. Muscadine grape extract as a potential treatment for prostate cancer

Prostate cancer remains a global health concern, driving the exploration of alternative therapeutic approaches to improve outcomes, especially for individuals diagnosed with biochemically recurring (BCR) prostate cancer. MSKE and resveratrol from red grapes have displayed the potential to inhibit prostate cancer development through distinct mechanisms. These investigations examined the effects of MSKE and resveratrol on prostate cancer cells, elucidating their potential as therapeutic agents. The investigations above used primary cultures of normal prostate epithelial cells (PrEC) and a range of prostate cancer cell lines representing different stages of prostate cancer progression.

For example, Paller et al. (2018) conducted a clinical trial involving prostate cancer patients to evaluate MuscadinePlus (MPX), a commercial product of pulverized muscadine grape skin, as a treatment option (Paller et al., 2018). This 12-month, multicenter, placebo-controlled study aimed to detect differences in PSA doubling time (PSADT) in 125 men with BCR prostate cancer low dose (500 mg) and high dose (4,000 mg) MPX compared to placebo. While the study revealed no significant difference in PSADT change between control and treatment groups, a subgroup analysis suggested potential benefits for patients with a specific genetic makeup. The administration of MPX to individuals with the SOD2 Alanine/Alanine genotype ((rs4880 T > C polymorphism) resulted in a significant increase in PSADT.

In another study, Hudson et al. (2007) conducted in vitro tests to explore the inhibitory effects of MSKE on tumor cell proliferation, which were found to be exceptional (Hudson et al., 2007). MSKE exhibited significant inhibitory effects on all prostate cancer cell lines tested, while normal PrEC cells did not respond similarly. The observed differential response highlights the selectivity of MSKE in targeting cancer cells. Moreover, the study reported an increased apoptosis rate in prostate tumor cell lines following treatment with MSKE. Apoptosis was induced by targeting key survival pathways, including phosphatidylinositol 3-kinase–Akt and mitogen-activated protein kinase. The study demonstrated that MSKE could decrease Akt activity through various mechanisms. These mechanisms include suppressing the transcription of Akt, promoting proteasome-mediated degradation of Akt, and modulating the levels of DJ-1, a known regulator of PTEN. These findings suggest MSKE has the potential to induce apoptosis in prostate cancer cells by disrupting critical pathways crucial for cell survival.

In contrast, Sweeney et al. (2021) found that resveratrol did not induce apoptosis in the prostate cancer cell model, differing from the effects observed with MSKE. However, resveratrol arrested the cells at the G1-S phase transition of the cell cycle, an effect linked to increased expression of p21, a cell cycle inhibitor, and reduced expression of cyclin D1 and cyclin-dependent kinase four proteins. This highlights resveratrol’s distinctive mode of action compared to MSKE, particularly in cell cycle control.

Subsequent investigations focused on the clinical assessment of MPX in men diagnosed with BRPC, a cohort of patients actively seeking alternate treatments to androgen restriction therapy (Paller et al., 2018). The first phase aimed to assess the safety, tolerability, and optimal dosage of MPX in BRPC patients, with increasing dosages of MPX found to be well tolerated. The maximum dosage tested, 4,000 mg/d, was deemed safe for further investigation, with no significant adverse effects noted. The second phase, a randomized, multicenter, placebo-controlled trial, evaluated the efficacy of MPX in these patients. While the study did not find a statistically significant difference in PSADT lengthening between the control and treatment groups, it did reveal potential benefits for a subset of patients with specific genetic characteristics. The SOD2 Alanine/Alanine genotype (Paller et al., 2015).

Burton and colleagues conducted a comprehensive investigation of the effect of MSKE on prostate cancer cells (Burton et al., 2014). The study proposed the hypothesis that MSKE could induce endoplasmic reticulum (ER) stress, leading to unfolded protein response (UPR)-mediated autophagy, ultimately resulting in prostate cancer cell death. To test this theory, they administered MSKE to C4–2 prostate cancer cells and conducted a quantitative proteome analysis. The results revealed a significant increase in the expression of proteins associated with the ER stress response, particularly glucose-regulated protein 78 (GRP78). These findings suggest that MSKE induces ER stress response in prostate cancer cells, leading to subsequent apoptosis. Western blot analysis confirmed elevated pro-apoptotic markers and reduced expression of anti-apoptotic markers.

Furthermore, MSKE treatment increased acridine orange and LC3B staining, providing evidence of autophagy in the treated cells. These observations suggest that MSKE has the potential to induce apoptosis by triggering autophagy through endoplasmic reticulum (ER) stress. Furthermore, this study focused on the transcription factor Snail and its role in epithelial-mesenchymal transition (EMT), which is crucial in cancer cells (Burton et al., 2014). This research explored Snail EMT in prostate cancer cells, focusing on the role of reactive oxygen species (ROS), particularly superoxide. Overexpressed Snail led to increased levels of mitochondrial superoxide. Notably, MSKE therapy reduced superoxide levels in these cells and effectively reversed the EMT process in cells overexpressing Snail. This reversal was concomitant with reduced vimentin levels and the restoration of E-cadherin expression, suggesting the shift back to an epithelial phenotype. MSKE also reduces cell migration, indicating its potential in suppressing cancer cell metastasis. In additional studies, Burton et al. investigated the effects of MSKE on bone turnover in prostate and breast cancer cells exhibiting Snail overexpression (Burton et al., 2015). Snail overexpression was associated with increased expression and activity of Cathepsin L (CatL) and phosphorylation of STAT-3 (pSTAT-3), both linked to cancer progression. MSKE treatment decreased Snail and pSTAT3 expression and acted as an antagonist to Snail-induced CatL activity. It also significantly inhibited Snail-induced osteoclastogenesis, a process associated with bone metastases.

These findings highlight the diverse mechanisms through which MSKE could potentially impede cancer growth and metastasis (Table 3). Further research and comprehensive clinical studies are imperative to explore MSKE’s potential as an all-natural compound with anti-cancer properties, particularly in metastatic diseases and the regulation of bone turnover.

## 7. Mechanisms of action in cancer cells

The anti-cancer properties of MGSE are facilitated through various mechanisms. One of the mechanisms that have been extensively investigated involves the modulation of signaling pathways associated with cell survival and proliferation. To examine the impact of MGSE on tumor development, nude mice bearing human MDA-MB-231 triple-negative breast cancer (TNBC) tumors were administered a proprietary Muscadine Grape Extract (MGE) for four weeks to assess tumor development. The results showed reduced tumor volume and decreased Ki67 and cyclin D1 (Collard et al., 2020). Further examination of the molecular mechanisms behind MGE-induced tumor growth reduction involved the treatment of mouse 4 T1, MDA-MB-231, and human BT-549 TNBC cells with MGE, with a focus on the associated signaling pathways. MGE inhibited c-Met, ERK/MAPK, AKT signaling, and cyclin D1, a downstream target of these pathways, leading to retinoblastoma stimulation and subsequent cell cycle arrest in MDA-MB-231 cells. Notably, TNBC cells are known for reduced cyclin D1 expression. The observed dose-dependent decrease in cell proliferation was attributed to MGE-regulated molecular signaling pathways. The versatility of MGE, coupled with its exceptional safety and tolerance, suggests its potential to halt the progression of TNBC into metastatic disease (Collard et al., 2020).

**Fig. 2.**
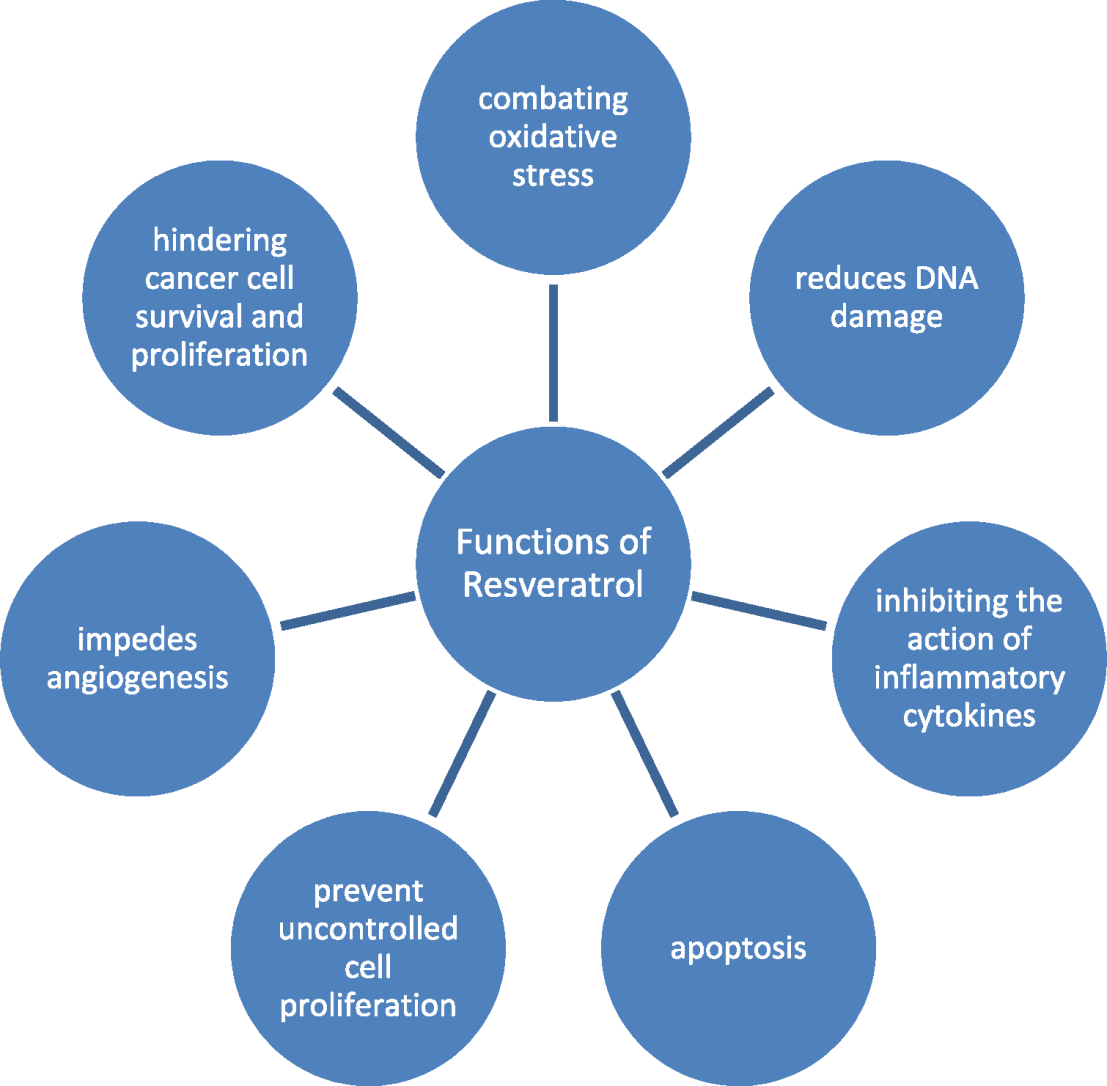
Functions of resveratrol in cancer management.

Resveratrol has emerged as a promising candidate in cancer management due to its multifaceted mechanisms of action (Fig. 2). This polyphenolic compound exerts various effects that contribute to its potential anti-cancer properties.

Among its primary roles, resveratrol is an antioxidant that combats oxidative stress and reduces DNA damage, and it is a recognized cancer instigator (Pervaiz & Holme, 2009). Moreover, it demonstrates potent anti-inflammatory properties by inhibiting the action of inflammatory cytokines and enzymes, which play pivotal roles in cancer progression (Alarcón de la Lastra & Villegas, 2005). Resveratrol controls the cell cycle, halting it at critical checkpoints to prevent uncontrolled cell proliferation while promoting programmed cell death, or apoptosis, in cancer cells. Additionally, it impedes angiogenesis, forming new blood vessels that supply tumors, thereby curbing tumor growth and metastasis(Alarcón de la Lastra & Villegas, 2005; Pervaiz & Holme, 2009).

Depending on the context, resveratrol can activate and inhibit pathways, including PI3K/Akt, NF-κB, and Wnt, thus hindering cancer cell survival and proliferation. Resveratrol’s epigenetic modifications and hormone regulation effects are also notable, potentially reprogramming cancer cells and sensitizing them to conventional therapies. It may improve the efficacy of chemotherapy and radiation therapy, making treatment more effective (Alarcón de la Lastra & Villegas, 2005; Fakhri et al., 2022). While promising, it is crucial to acknowledge that resveratrol’s effectiveness can vary by cancer type and other factors. Further clinical research is needed to fully understand its potential and safety as an adjuvant in cancer therapy. Nonetheless, resveratrol’s versatile mechanisms of action offer valuable insights into innovative strategies for cancer management.

Similarly, the tannins found in MGSE provide a multifaceted approach to combating cancer. Their prominent role lies in their potent antioxidant properties, guardians against harmful free radicals and oxidative stress, and are known culprits in DNA damage and cancer initiation (Yi et al., 2005). By reducing oxidative damage, MGSE tannins can potentially thwart the early stages of cancer development. Furthermore, these tannins possess anti-inflammatory attributes, another critical facet of cancer prevention and treatment (Baer-Dubowska et al., 2020). Chronic inflammation is closely associated with cancer progression (Qian et al., 2019), and tannins’ anti-inflammatory effects can help mitigate this environment, inhibiting the growth and spread of cancer cells (Y. Li et al., 2021). Tannins are also adept at regulating the cell cycle, ensuring that cell division remains in check.

Similarly, as a natural chemical, tannins/Tannic acid (TA) plays a vital function in each transition phase in the process of carcinogenesis (Pinakin et al., 2020). It has been reported to result in cell cycle arrest, apoptosis induction, decreased cell proliferation, migration, and reduced adhesion in many cancer cell lines, primarily by controlling various signaling pathways, including EGFR/Jak2/STATs, TGF-β, and NF-Kβ, or suppressing the PKM2 glycolytic enzyme. Youness et al. (2021) documented significant anti-cancer properties of TA against many solid tumors, such as lung, breast, ovarian, and colorectal cancer. The impact of TA on many oncological signal pathways, including VEGF/VEGFR, RAS/RAF/mTOR, JAK/STAT, TGF-β1R/TGF-β1R axis, and CXCL12/CXCCR4 axes, has been seen. TA can initiate endoplasmic reticulum (ER) stress through the unfolded protein response (UPR) pathway, leading to the activation of apoptosis as a potential strategy for cancer treatment (Yang et al., 2021). Cell death and apoptosis in cancer cells are accelerated by ER stress [70], as ER is responsible for critical processes, including protein synthesis, maturation, folding, and transport (Lemmer et al., 2021). TA’s impact on cell viability is attributed to its efficient regulation of lipid metabolism and its ability to disrupt both cell and nuclear membranes. TA is exceptionally skilled at targeting lipids and generating ER stress through reactive oxygen species (ROS), as demonstrated in a study on prostate cancer [72]. Prostate cancer cells use abnormal lipid signaling and metabolism to enhance their survival capabilities. TA’s capacity to disrupt lipid signaling and suppress lipid metabolic pathways modulates the equilibrium between cell survival and death (Nagesh et al., 2020).

Furthermore, Catechins are potent antioxidants that scavenge free radicals and minimize oxidative damage (Bawono et al., 2023). This vital process prevents DNA damage and mutations that might cause cancer. Catechins also regulate the cell cycle, ensuring controlled cell division, inhibiting cancer cell proliferation, and promoting apoptosis. A study by Sun et al. (2020) aimed to evaluate the anti-cancer efficacy and mechanism of catechin against A549 cells, a non-small cell lung cancer. At a concentration of 600 μmol·L − 1 and after 24 h of incubation, the inhibitory rate of catechin on the growth of A549 cells was found to be 19.76 %. According to the research results, catechin reduces the growth of malignant A549 cells by increasing their production of the proteins p21 and p27. In addition, the administration of catechin resulted in the suppression of cyclin E1 expression and protein kinase phosphorylation (P–AKT) in a way dependent on the dosage. This mechanism further contributed to the prevention of proliferation in cancer cells. These findings demonstrate that catechin exhibits a significant capacity to impede the growth of A549 cells by modulating their cell cycle arrest, either directly or indirectly, via the activation of the p21 signaling pathway. This research provides significant insights into the development of catechin and catechin-rich functional food or co-therapy for anti-cancer applications. However, the effects of catechins on cancer may vary based on the categories and quantities of catechins, as well as the type of cancer under investigation. Further research and clinical trials are required to fully harness catechins’ cancer-fighting potential.

**Fig. 3.**
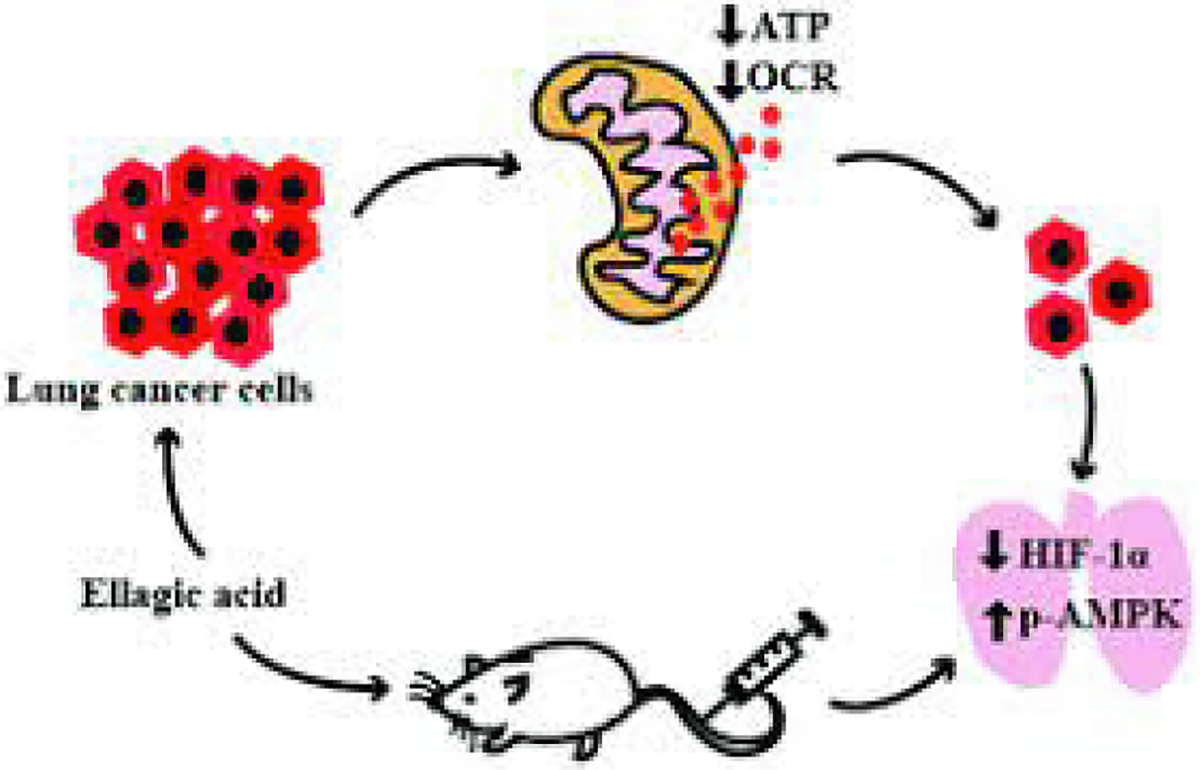
Ellagic acid’s mechanism of action in effectively suppressing lung cancer cell growth. According to Lee et al. [75].

Lastly, ellagic acid (EA) and gallic acids are two vital polyphenolic chemicals in MGSE (A. Marshall, 2012; Lee & Talcott, 2002). These compounds exhibit the potential to impede cancer growth through several mechanisms. Collectively, these effects highlight their significance in cancer prevention and therapy. These effects primarily include the suppression of tumor development through the inhibition of cell proliferation, induction of apoptosis, and impairment of mitochondrial function. A study by Lee et al. (Lee & Talcott, 2002) found that EA effectively suppressed the growth of lung cancer cells (Fig. 3). Moreover, it significantly reduced ATP levels, diminished the potential of the inner mitochondrial membrane, and lowered oxygen consumption in an in vitro setting. EA’s activation of AMP-activated protein kinase (AMPK) reduced HIF-1α levels in lung cancer cells. Additionally, administering EA to mice with tumors significantly impeded tumor development, elevated p-AMPK levels, and reduced the HIF-1α levels. These results indicate that EA may be a chemotherapeutic drug that specifically affects mitochondrial metabolism in lung cancer.

Nonetheless, ongoing research is essential to explore these treatments’ effectiveness further, considering variables such as concentration levels and specific cancer types. More extensive research, including clinical trials, is imperative to harness these substances’ medicinal potential fully.

## 8. Combinatorial approaches to conventional treatments

The concept of synergy between MGSE and traditional cancer treatments has attracted considerable attention within the dynamic field of cancer therapy. Integrating MGSE with established cancer therapies offers a promising avenue to enhance treatment outcomes, mitigate side effects, and elevate the overall quality of cancer care. For instance, the potential of MGSE to strengthen the efficacy of chemotherapy, a fundamental component of cancer therapy, has been the subject of investigation (Kaur et al., 2009). Research has demonstrated that MGSE has the potential to enhance the sensitivity of cancer cells toward chemotherapy agents, thereby augmenting their vulnerability to therapeutic intervention. This paves the way for achieving therapeutic goals with reduced chemotherapy dosages, thereby minimizing adverse effects (Kaur et al., 2009; Patra et al., 2021).

*Radiation therapy* plays a pivotal role in cancer treatment, and efforts have been made to explore the combination of MGSE and radiation to amplify the effectiveness of radiation therapy (Abshire & Lang, 2018). A study by Chen et al. reported the effects of Muscadine grape extract on inhibiting brain metastasis in breast cancer cells (Chen et al., 2017). The study also assessed the effectiveness of co-administering MGE and ionizing radiation (IR) in terms of therapeutic response. Although MGE alone did not sensitize cells to IR, the concurrent administration of MGE and IR significantly reduced clonogenic survival compared to using either modality individually. This effect was consistent across both cell lines and was supported by numerous tests (n = 3–4 experiments, p < 0.01). These findings suggest an additive effect when MGE and IR are combined. MGSE’s ability to regulate signaling pathways and impede tumor proliferation complements the direct cytotoxic effects of radiation therapy.

*Immunotherapy* has significantly transformed cancer treatment by harnessing the immune system to selectively target malignant cells (Peterson et al., 2022). The immunomodulatory properties of MGSE render it a compelling candidate for integration with immunotherapeutic approaches. The potential of MGSE to augment the efficacy of immunotherapeutic agents lies in its ability to stimulate immune responses against cancer cells. Bitting et al. conducted a Phase I study on using MGE for patients diagnosed with advanced cancer (Bitting et al., 2021). They observed that over 60 % of patients, particularly those with extensive prior treatments, participated in the trial for at least eight weeks. Notably, an 84-year-old patient with lung adenocarcinoma, who had opted for a focus on quality of life over cytotoxic therapy due to toxicity, experienced prolonged disease stability exceeding two years under MGE therapy.

*Hormone therapy* is a widely employed treatment modality for hormone-responsive malignancies, including breast and prostate cancer (Lyman, 2015). MGSE’s potential to exhibit anti-inflammatory and anti-proliferative properties could potentially enhance the efficacy of hormone therapy by reducing inflammation and inhibiting cancer cell proliferation. This combined approach shows promise for improving the effectiveness of hormone therapy interventions. Targeted therapies prevent cancer cell growth and spread by inhibiting specific molecules (Wabel & Khajah, 2019). MGSE’s capacity to regulate signaling pathways renders it a prospective candidate for integration with targeted therapeutic approaches. Collectively, these methodologies have the potential to offer a multifaceted strategy for targeting cancer cells.

The era of personalized medicine in cancer care recognizes the uniqueness of each patient’s cancer. Combinatorial approaches using MGSE can be tailored to individual patients, taking into account the specific characteristics of their cancer, thereby enabling a more accurate and efficient treatment approach. The incorporation of MGSE into combinatorial methodologies alongside conventional treatments presents a potentially fruitful avenue in the field of cancer therapy. These approaches can potentially enhance treatment, alleviate side effects, enhance patients’ overall well-being, and expand the range of therapeutic options accessible to individuals facing cancer.

## 9. Anticancer Potential: Muscadine grape Skin extract vs other plant extracts

Throughout history, using plant-derived substances has been pivotal in cancer treatment. Hence, exploring phytochemicals has led to the discovery of effective anticancer medications. Numerous plant species, such as the *Azadirachta indica* (Moga et al., 2018) and *Morinda citrifolia* (Chanthira Kumar et al., 2022), have shown the presence of anti-cancer capabilities in their respective floral components. Pomegranate (Panth et al., 2017), brassinosteroids (Panibrat et al., 2019), medicinal mushrooms (De Silva et al., 2012), ginger extract (de Lima et al., 2018), and turmeric extract (Unlu et al., 2016), have been shown to possess qualities that might inhibit the growth and spread of cancer cells.

However, MGSE distinguishes itself from Curcumin because it possesses many phytochemicals, such as resveratrol, quercetin, and ellagic acid, contributing to their distinctive composition surpassing a single active component Curcumin turmeric possesses (God et al., 2007; Unlu et al., 2016). Furthermore, ongoing research is being done to investigate the safety profile and toxicological features of MGSE, which has shown encouraging results. Although Curcumin is known to have difficulties with bioavailability, ongoing research is focused on developing methods to improve the absorption of MGSE, which may help overcome this restriction (Stewart et al., 2023; Yuzuak & Xie, 2022).

Therefore, MGSE is notable for its unique composition of polyphenols, stilbenes, and antioxidants, distinguishing them as a prominent category of anti-cancer plants. These extracts provide a diverse array of therapeutic benefits. The efficacy of these fruits in combating cancer may be attributed to their elevated levels of phytochemicals such as resveratrol and ellagic acid. Scholars have focused on investigating the potential synergistic mechanisms by which stilbenes may enhance their cytotoxicity. The efficacy of muscadine grape extracts in combating several types of malignancies, including prostate and breast cancer, demonstrates its remarkable versatility. It is noteworthy to acknowledge that the impact of these substances extends beyond the inhibition of tumors. Thus, MGSE stands out among various plant extracts, significantly contributing to cancer research.

## 10. Safety and side effects

Exploring the therapeutic potential of MGSE in cancer treatment and prevention necessitates a thorough, comprehensive understanding of its safety profile and possible side effects. Toxicology studies involving animal subjects have evaluated the effects of MGSE on various organs and systems through toxicology studies that utilize animals as subjects (Paller et al., 2015, 2018). These studies consistently report a favorable safety profile for the product, with no significant adverse effects observed at the recommended doses (Hudson et al., 2007). This reassuring evidence has facilitated the progression of additional research involving human subjects. Early-phase clinical trials have primarily focused on assessing the safety and tolerability of MGSE in humans. These trials have recorded minimal adverse effects, mainly limited to mild gastrointestinal discomfort experienced by specific individuals. Notably, severe adverse events associated with MGSE have been rare. Understanding dose–response relationships is imperative to ensure the safe use of MGSE, as it provides insights into how the effects of MGSE may vary in dosage. Toxicology studies and dose-escalation trials have played a crucial role in determining appropriate dosage ranges for MGSE, guiding clinical protocols, and mitigating the risk of potential toxic effects (Hudson et al., 2007; Paller et al., 2018). The phytochemicals present in MGSE have the potential to interact with specific medications, influencing their absorption or metabolism (Y. Huang et al., 2021). It is imperative for individuals undergoing cancer treatment to seek guidance from healthcare professionals before integrating MGSE into their treatment plan. Data regarding the safety of MGSE during pregnancy and lactation are limited. Pregnant and breastfeeding individuals should exercise caution and seek guidance from healthcare professionals before use (Jamal, 2023).

## 11. Future perspectives

Incorporating MGSE into conventional cancer treatment presents several challenges and unanswered questions. A significant factor in determining the optimal MGSE dosage for multiple cancer types and stages presents a multifaceted challenge. The perpetual challenge lies in balancing treatment’s therapeutic efficacy and potential adverse effects mitigation (Lee & Talcott, 2002). While short-term studies have indicated favorable safety profiles, additional research is needed to examine the prolonged impact of MGSE supplementation, particularly in individuals who have survived cancer. Understanding the molecular pathways through which MGSE exerts its anti-cancer effects is crucial, and this may be achieved through gaining mechanistic insights. Moreover, variability in the formulations and concentrations of MGSE across different products can present research and clinical challenges. Standardization efforts are essential to ensure uniform quality and effectiveness (Apostolaros et al., 2020).

## 12. Conclusion

The use of MGSE holds significant potential in the ongoing battle against cancer. An exploration of the research landscape reveals its diverse range of possibilities. In vitro investigations have elucidated the complex anti-cancer mechanisms of MGSE, while clinical trials have confirmed its safety and provided opportunities for further exploration of its efficacy. Integrating combinatorial strategies with conventional therapies presents promising prospects for advancing comprehensive cancer treatment. The versatility of MGSE is evident across a wide range of cancer types, suggesting its potential as a complete solution. The extensive implications of its role as an adjuvant therapy, cancer prevention agent, and cornerstone of personalized medicine are noteworthy. Incorporating MGSE within the framework of patient-centered care enhances individuals’ autonomy and agency throughout their therapeutic trajectories.

Hence, it becomes evident that MGSE’s lasting capacity to revolutionize cancer care is apparent. The symbolization of renewed hope for patients and families globally indicates a forthcoming era with the potential for innovative therapies derived from natural resources, offering the prospect of more optimistic futures. With each discovery, we progress towards fully harnessing the potential of MGSE, which presents itself as a formidable tool in the ongoing fight against cancer.

## Figures and Tables

**Table 1:** Notable Phytochemical Compounds in Muscadine Grape Skin Extract (MGSE) and Their Health Benefits.

Phytochemical Compounds	Function	References

Catechins, Epicatechin, Gallic acid	Antioxidant, Potential anticancer properties, Supports Cardiovascular Health, Antiinflammatory, Antioxidant.	(Alrugaibah et al., 2021; Yilmaz & Toledo, 2004)
Stilbene, Ellagic Acid	Anti-inflammatory, antioxidant, slows cancer cell growth, Induces apoptosis (programmed cell death)	(A. Marshall, 2012)
Resveratrol	A powerful antioxidant with the potential to inhibit cancer cell proliferation, Cardio-protective properties	(Balasubramani et al., 2019; Hudson et al., 2007)
Quercetin, Myricetin, kaempferol	A potent antioxidant, Reduces the risk of certain cancers and Regulates cancer cell proliferation and viability.	(Pastrana-Bonilla et al., 2003)
Anthocyanins, Proanthocyanidins	Antioxidant, Anti-inflammatory, inhibits tumor growth, Impedes angiogenesis (formation of new blood vessels)	(Ma & Zhang, 2017)
Tannins	Antioxidants, Possible anticancer properties, May help control blood sugar levels.	(Sandhu & Gu, 2010)

**Table 2:** Comparison of the Anticancer potentials of Muscadine Grape Skin Extract and other Plant-Derived medicinal extracts.

Anti-cancer Compounds	Muscadine Grape Skin Extract	Turmeric (Curcumin)	Green Tea	Ginseng	Garlic	Ginger

Allicin	—	—	—	—	Yes	—
Alliin	—	—	—	—	Yes	—
Anthocyanins,	Yes	—	—	—	—	—
Catechins,	Yes	—	Yes	—	—	—
Curcumin		Yes		—	—	—
EGCG (Epigallocatechin gallate)	—	—	Yes	—	—	—
Ellagic Acid	Yes	—	—	—	—	—
Ellagitannins	Yes	—	—	—	—	—
Epicatechin,	Yes	—	—	—	—	—
Gallic acid	Yes	—	—	—	—	—
Gingerol	—	—	—	—	—	Yes
Ginsenosides	—	—	—	Yes	—	Yes
kaempferol	Yes	—	—	—	—	—
Myricetin,	Yes	—	—	—	—	—
OPCs (Oligomeric Proanthocyanidins)	Yes	—	—	—	—	—
Proanthocyanidins	Yes	—	—	—	—	—
Quercetin	Yes	—	—	—	—	—
Resveratrol	Yes	—	—	—	—	—
Shogaol	—	—	—	—	Yes	—
Stilbene	Yes	—	—	—	—	—
Tannins	—	—	—	—	—	—

**Table 3:** Summary of Muscadine Grape ski Extract on cancer research.

Author	Cancer Type Studied	Aim	Dosage	Treatment Duration	Models	Human Equivalent Doses	Key Findings and Implications

Mendonca et al., 2019a	Triple-negative breast cancer (TNBC)	Investigate anticancer and antioxidant effects	400 μg/ reaction (4 μg/μl)	24 h	In vitro (cell culture)	2.8 mg/ml	- Muscadine grape extract showed high antioxidant and anticancer activity, particularly in African American breast cancer cells.
Collard et al., 2020	Triple-negative breast cancer (TNBC)	Explore effects on breast cancer cell migration	0.5 mg total phenolics/ mouse/day for a 25 g mouse	Four weeks	6-week-old female athymic mice	20 μg /mL	- Suggested its potential as an antioxidant and anti-cancer agent for TNBC.
Collard et al., 2019	Triple-negative breast cancer (TNBC)	Inhibit TNBC metastasis and alter the gut microbiome	0.1 mg/mL	Four weeks	6-week-old female Balb/c mice	Not specified	- MGE effectively inhibited TNBC tumor growth and reduced key tumor markers.
Burton et al., 2019	Triple-negative breast cancer (TNBC)	To examine the involvement of CUX1, a downstream substrate of Cat L, in triple-negative breast cancer (TNBC).	20 μg/mL	24 h	The human breast cancer cells lines MCF10-A, MCF-7, and MDA-MB-468, and MDA-MB-231	20 μg/mL	- Suggested its potential as a preventive or therapeutic agent for TNBC, possibly in combination with other treatments.
Mackert et al., 2019	Breast cancer	To evaluate the potential inhibitory effects of MGE on the growth of both TRZ- sensitive and -resistant HER2 positive breast cancer cells.	20 μg/mL	24 h	HER2-positive breast cancer cells	20 μg/mL	- MGE reduced lung and liver metastasis in TNBC mice.
Balasubramani et al., 2019b	lung carcinoma cells, triple-negative breast cancer, and liver cancer cells	To assess the anticancer efficacy of muscadine berry extract, which is high in stilbenes and pure resveratrol.	1 μg/mL	72 h	A549 (lung carcinoma cells), triple-negative breast cancer (HCC- 1806), and HepG2 (human liver cancer) cells	Not specified	- Altered gut microbiome composition and increased butyrate-producing bacteria, suggesting potential as a TNBC metastasis treatment.
Hudson et al., 2007	Prostate cancer	to assess and compare the antitumor effects of MGSE devoid of resveratrol with those of resveratrol itself.	0, 2, 5, 10, 20 μg/mL) was given daily	24, 48, and 72 h	prostate cancer cell lines RWPE-1, WPE1- NA22, WPE1-NB14, and WPE1-NB26	Not specified	- CUX1 was found to repress ER-α in TNBC cells, promoting TNBC progression.
Paller et al., 2015	Prostate cancer	To investigate the safety, acceptability, and dosage determination of MGSE in males with biochemically recurrent prostate cancer via a phase I clinical trial.	500 to 4,000 mg of MPX	15 months	men with biochemically recurrent prostate cancer	4,000 mg	- MSKE suppressed CUX1 and restored ER-α expression, inhibiting cell invasion and migration.
Paller et al., 2018b	Prostate cancer	To assess the potential therapeutic benefits of MuscadinePlus (MPX), a commercially available product made from powdered muscadine grape peel, for men with biochemically recurring (BCR) prostate cancer who choose to delay androgen deprivation treatment.	500 mg MPX (low) and 4,000 mg MPX (high)	Six months (low dose) and 12 months (high dose)	Men with biochemically recurrent (BCR) prostate cancer	Not specified	- Suggested MSKE as a potential TNBC therapeutic targeting CUX1.
Burton et al., 2016	Prostate Cancer	Investigate MGSE’s effects on breast cancer	20 μg/ml	72 h	C4–2 human prostate cells	20 μg/mL	- MGE effectively inhibited HER2-positive breast cancer cell proliferation, including resistant cells.
Burton et al., 2014	Prostate cancer	Study MGSE’s impact on colon cancer	5 μg/ml	Three days	ARCaP and LNCaP prostate cancer cells	5 μg/ml	- Suggested potential therapeutic use of MGE alone or in combination with trastuzumab for HER2-positive breast cancer.

## Data Availability

No data was used for the research described in the article.
